# Multivariate decoding of cerebral blood flow measures in a clinical model of on‐going postsurgical pain

**DOI:** 10.1002/hbm.22652

**Published:** 2014-10-12

**Authors:** Jonathan O'Muircheartaigh, Andre Marquand, Duncan J. Hodkinson, Kristina Krause, Nadine Khawaja, Tara F. Renton, John P. Huggins, William Vennart, Steven C.R. Williams, Matthew A. Howard

**Affiliations:** ^1^ Department of Neuroimaging Institute of Psychiatry, Centre for Neuroimaging Sciences, King's College London London United Kingdom; ^2^ Donders Institute for Brain Cognition and Behaviour, Radboud University Nijmegen The Netherlands; ^3^ Department of Oral Surgery, King's College London Dental Institute London United Kingdom; ^4^ Pfizer Global Research and Development Sandwich United Kingdom

**Keywords:** pain, biomarker, arterial spin labeling, machine learning

## Abstract

Recent reports of multivariate machine learning (ML) techniques have highlighted their potential use to detect prognostic and diagnostic markers of pain. However, applications to date have focussed on acute experimental nociceptive stimuli rather than clinically relevant pain states. These reports have coincided with others describing the application of arterial spin labeling (ASL) to detect changes in regional cerebral blood flow (rCBF) in patients with on‐going clinical pain. We combined these acquisition and analysis methodologies in a well‐characterized postsurgical pain model. The principal aims were (1) to assess the classification accuracy of rCBF indices acquired prior to and following surgical intervention and (2) to optimise the amount of data required to maintain accurate classification. Twenty male volunteers, requiring bilateral, lower jaw third molar extraction (TME), underwent ASL examination prior to and following individual left and right TME, representing presurgical and postsurgical states, respectively. Six ASL time points were acquired at each exam. Each ASL image was preceded by visual analogue scale assessments of alertness and subjective pain experiences. Using all data from all sessions, an independent Gaussian Process binary classifier successfully discriminated postsurgical from presurgical states with 94.73% accuracy; over 80% accuracy could be achieved using half of the data (equivalent to 15 min scan time). This work demonstrates the concept and feasibility of time‐efficient, probabilistic prediction of clinically relevant pain at the individual level. We discuss the potential of ML techniques to impact on the search for novel approaches to diagnosis, management, and treatment to complement conventional patient self‐reporting. *Hum Brain Mapp 36:633–642, 2015*. © **2014 The Authors. Human Brain Mapping Published by Wiley Periodicals, Inc**.

**Abbreviations**
ASL: Arterial Spin Labeling
fMRI: Functional Magnetic Resonance Imaging
GP: Gaussian Process
GPC: Gaussian Process Classification
LOOCV: Leave‐one‐out Cross Validation
ML: Machine Learning
MPC: Multivariate Pattern Classification
PET: Positron Emission Tomography
rCBF: Regional Cerebral Blood Flow
TME: Third Molar Extraction

## INTRODUCTION

Despite decades of effort and investment, effective pain management remains an unmet need worldwide [Kupers and Kehlet, 2006]. New therapies must be developed to help bridge this gap [Woolf, 2010], but current clinical trials continue to utilise self‐report as their sole endpoint to assess success. This reliance on self‐report to record individuals' pain experiences is understandable given the absence of a known biological index of pain. Improving pain phenotyping and developing more sensitive measurement techniques should add value to self‐report [Robinson et al., 2013]. Ideally such new methods would also provide robust, individualized predictions of treatment response [Rosa and Seymour, 2014; Woodcock et al., 2007].

Neuroimaging has sought to address these aspirations but there have been significant limitations in its application to the study of on‐going or “background” pain, often a defining feature of clinical pain disorders [Kupers and Kehlet, 2006]. On‐going pain is difficult to measure using conventional functional magnetic resonance imaging (fMRI) methods, such as blood oxygen level‐dependent (BOLD) imaging [Downar et al., 2000; Legrain et al., 2011], as this behavior cannot be broken up into blocks or interrupted by rest. Positron Emission Tomography does not suffer this limitation but is more expensive, less accessible, and constrained by safety concerns regarding repeat‐administration of radioactive ligands as required in “cross‐over” studies. Resting‐state fMRI offers promise but the mechanisms underlying correlations between brain regions during the pain experience remain poorly understood [Napadow et al., 2010]. Another MRI‐based technique, arterial spin labeling (ASL), provides noninvasive, quantitative indices of cerebral blood flow (CBF) with the sensitivity to detect “tonic” states over the course of minutes [Aguirre et al., 2002]; thus, ideally suitable for examining persistent or on‐going pain. Our own preliminary research [Hodkinson et al., 2013; Howard et al., 2011] has documented the feasibility and reliability of the third molar tooth extraction (TME) model to study on‐going postsurgical pain using ASL. Pain intensity following TME has been extensively evaluated and shown to produce moderate‐to‐severe on‐going pain between 3 and 5 hrs following surgery [Barden et al., 2004].

Recently (see [Rosa and Seymour, 2014] for a review), neuroimaging analyses have taken advantage of the richness of information that is available within fMRI data to decode the pain experience. Multivariate analyses acknowledge spatial relatedness in whole‐brain imaging datasets, offering greater sensitivity than conventional mass‐univariate methods to detect spatially distributed effects [Norman et al., 2006]. Supervised “machine learning” (ML) pattern classifiers potentially offer the desirable quality of predicting class membership of new individuals, for example, whether a new patient is in pain or might respond to treatment [Marquand et al., 2012]. Despite their impact in the field to date, ML techniques have yet to have been applied to the critical challenges of on‐going clinical pain. Here, we address this issue directly, applying Gaussian Process Classification (GPC) to a pre‐existing clinical ASL dataset [Howard et al., 2011]. GPC yields similar accuracy to other ML techniques such as Support Vector Machines [Marquand et al., 2010] but provides the advantage of probabilistic predictions that can capture variability within clinical populations. The two principal aims of this study were: (i) to determine GPC accuracy in discriminating presurgical from postsurgical states, following left and right TME; (ii) to understand the temporal effects of acquiring multiple ASL scans on this classification accuracy.

## MATERIALS AND METHODS

### Participants

Twenty right‐handed, healthy, male volunteers aged 20–34 (mean age = 26.18 years) provided written, informed consent to participate in the study. Female participants were excluded due to possible variability induced by the phase of the menstrual cycle on postsurgical pain [Teepker et al., 2010]. All participants presented with bilateral recurrent pericoronitis and fulfilled NICE (2000) guidelines for extraction of lower‐jaw left and right third molars. The study was approved by King's College Hospital NHS Research ethics committee (07/H0808/115).

### Experimental Design

**Figure 1:**
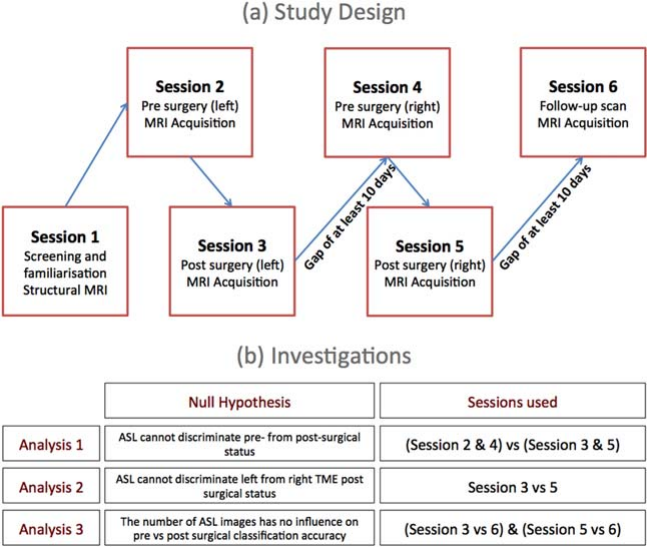
Study design illustrating the order of visits for each participant (**a**). The three main investigations performed in this study as well as the datasets used for each are also indicated (**b**). Note that, although the order of surgery was pseudorandomized, in this figure Session 3 indicates the session acquired after left‐sided third molar extraction (TME), Session 5 right‐sided TME. [Color figure can be viewed in the online issue, which is available at http://wileyonlinelibrary.com.]

Participants visited on six separate occasions (S1–S6); screening/familiarisation (S1), presurgical (S2), and postsurgical sessions (S3) for the first extraction, presurgical (S4) and postsurgical (S5) sessions for the second extraction, and a final follow‐up session (S6). A minimum ten day interval separated S3/S4 and S5/S6, assuring complete recovery from each surgery. The order of left and right tooth extraction was balanced and pseudorandomized across the group. The study design is illustrated in Figure 1a. Patients' vitals were recorded before each session (i.e., pulse rate and blood pressure), in addition to an alcohol/drug‐screen and a psychometric assessment (the reader is directed to [Howard et al., 2011] for further detail on design and psychometry). Analgesic medication (1000 mg paracetamol and 400 mg ibuprofen) was available to participants immediately only following postsurgical scanning.

### Imaging Procedure

Imaging was performed on a 3 Tesla Signa HDx whole‐body MR imaging system (General Electric) fitted with an 8‐channel, phased‐array receive only head‐coil. High‐resolution *T*_1_‐ and *T*_2_‐weighted MR structural sequences were acquired in Session 1 for radiological assessment and image registration. In Sessions 2–6, resting‐state rCBF measurements were made using psuedocontinuous ASL (ASL) [Dai et al., 2008], using a labeling duration of 500 μs, peak to peak gap of 1,500 μs and a total labeling duration of 1.5 s. After a postlabeling lag of 1.5 s, images were acquired using a 3D Fast Spin Echo spiral readout sequence (8 shots, TE/TR 32/5500 ms. ETL = 64, 3 tag control pairs). Images were acquired over a 18 × 24 × 18 cm field of view with a 48 × 64 × 60 matrix, reconstructed to a nominal spatial resolution of 1 × 1 × 3 mm. The imaging protocol was identical at each session (S2–S6). Each of the 6 pCASL scans per session took approximately 6 min 8 s, so the total MRI session lasted approximately 40 min with no breaks. Patients were required to lie still with their eyes open during scanning.

### Visual Analogue Scales

During MRI acquisition in Sessions 2–6, a computerized visual analogue scale (VAS) recorded patient's subjective pain and alertness prior to the first ASL scan and then immediately following each subsequent scan, leading to 7 total VAS measurements for alertness and subjective pain per scanning session. The words “no pain”/“worst imaginable pain” and “very sleepy”/“wide awake,” respectively, were displayed as the left/right anchors, with visual feedback projected onto a screen located at the patient's feet and visible via a mirror.

### Image Preprocessing

Image analysis was carried out using a combination of tools from FSL v4.1.6 (http://www.fmrib.ox.ac.uk/fsl/) and SPM8 (v4010) toolkits (http://www.fil.ion.ucl.ac.uk/spm/). For each subject, all collected ASL images within and across sessions were coregistered with each other and a mean image generated [SPM]. The *T*_2_ weighted image was skull stripped using a brain extraction tool [FSL‐BET] and the resulting brain‐only image was coregistered with the average ASL image and used as a mask to exclude extra‐cerebral signal [SPM‐CO‐REGISTER]. A nonlinear transformation was calculated between the mean ASL image and a custom ASL template in the standardized, stereotaxic co‐ordinates of the Montreal Neurological Institute (MNI) [SPM‐NORMALISE]. The raw images were then transformed to MNI space in one interpolation step. The resulting images were smoothed with an 8 mm full width at half maximum isotropic Gaussian kernel. To account for the intersubject variability of global blood perfusion values, all normalized, smoothed images were scaled to have a median value of 1,000. This scaling was performed to ensure that global differences in CBF values did not confound later classification.

### Behavioral Data Analysis

VAS scores of perceived pain and alertness were averaged for each subject and session. Between‐session differences in pain and alertness were investigated using one‐way repeated measures ANOVA, with session (Left Postsurgery, Right Postsurgery, and Presurgery) used as the within‐subjects factor. Post‐hoc *t*‐tests were used to examine differences between individual sessions. Statistical analyses were performed using SPSS v18 (http://www.spss.com) and Microsoft Excel for Macintosh 2011 (http://www.microsoft.com/mac).

### Gaussian Process Classification

Independent binary GPCs [Rasmussen et al., 2006] were used to discriminate postsurgical from presurgical states, based on the whole‐brain ASL images. Three analyses were performed (see Fig. 1b for null hypotheses):

- discrimination of the postsurgical from presurgical state. To maximise sensitivity in this analysis, we used all presurgical (i.e., combined left and right images) and postsurgical ASL data from both third molar extractions (TME); 1One subject was excluded from this analysis due to the presence of an image artifact in ASL data acquired during S4.
- classification between left, compared to right‐sided postsurgical states;
- discrimination of the postsurgical from follow‐up pain‐free scans acquired 2 weeks following the second TME. We also examined the effects of using data from multiple ASL scans. On each side we then repeated the GPC analysis five times, progressively reducing the number of ASL scans available to train the classifier from six scans per session to one, performed in reverse acquisition order. As the purpose of this analysis was to determine the effect of the number of scans acquired on classification accuracy, the average of the remaining ASL images were used in both the training and testing phases.

#### Classifier implementation

A detailed description of the GPC approach used in this work has been provided elsewhere [Marquand et al., 2010; Rasmussen et al., 2006]. In brief, a separate GPC model was trained for each of the comparisons described above. At the core of each of these models were a set of latent function variables used to model the relationships between the data points. To model the categorical binary class labels, these variables were passed through a sigmoidal (probit) likelihood function, which has the effect of constraining them to lie in the unit interval. A Gaussian process prior with a linear covariance was then applied to the latent function and the posterior predictive distribution from this model was computed by applying the rules of probability calculus and integrating out the latent variables. These operations cannot be performed in closed form, so the expectation propagation algorithm was used to approximate the posterior distribution. Expectation propagation is well known to provide highly accurate estimates of the true posterior distribution and is the method of choice for GPC [Nickisch and Rasmussen, 2008]. Model hyperparameters controlling the scaling and bias of the latent function were optimized by maximising the model evidence, a procedure also referred to as type‐II maximum likelihood (see [Marquand et al., 2010] for full details). All GPC analyses were performed using the Probid software toolbox (http://www.brainmap.co.uk), which relies on the GPML toolbox for GPC inference (http://www.gaussianprocess.org/gpml).

#### Cross‐validation

All classifiers were embedded within a leave‐one‐out cross‐validation (LOOCV) framework to estimate the generalisation performance of each classifier for novel data points. This was achieved by repeatedly repartitioning the data into a test set (all scans from one subject) and a training set (all remaining scans) such that each subject was excluded once. Predictions derived from the test data were then used to compute the sensitivity and specificity of the classifier. These were defined respectively as the proportion of postsurgical or presurgical scans correctly classified across all LOOCV cycles. The sensitivity and specificity were then averaged to derive a balanced accuracy measure quantifying the overall performance of each classifier.

#### Significance testing

A permutation test was used to assess whether each classifier exceeded the accuracy that would be predicted by chance (50%). To achieve this, the entire LOOCV procedure was repeated 1,000 times after randomly permuting the class labels in a manner accommodating the repeated‐measures experimental design (i.e., labels were permuted at the subject level). The balanced accuracy was computed for each permutation and a *P*‐value for each classifier was derived by computing the proportion of random permutations achieving higher balanced accuracy than the nonpermuted classifier. To account for multiple comparisons in analysis (iii), the *P*‐values derived from the permutation test were corrected using Holm's Step‐Down procedure [Holm, 1979].

#### Visualising the classification pattern

For this application, it is desirable to know how blood flow distribution differs between experimental classes, so a mapping approach that enables direct visualization of the relative class distribution was used. Under this approach, the coefficient scores at each voxel represent the relative difference between experimental classes in the context of the entire pattern. They are constructed by projecting the training data onto the vector that defines the direction of maximal difference between experimental classes. In other words, this vector describes the mean of the posterior distribution in the input (i.e., voxel) space. Further details can be found in [Marquand et al., 2010]. It is important to note that these “g‐maps” differ from “w” or weight‐vector maps more commonly reported in multivariate neuroimaging analyses [Mourao‐Miranda et al., 2005] that illustrate the contribution of each brain region to the classifier decision. While “g‐maps” bear resemblance to conventional univariate *Z* or *T*‐statistical maps, it is important to reiterate that they should be interpreted as a pattern, rather than clusters of individual brain regions. As whole‐brain gray matter contributed to covariance estimates used by the classifier, g‐maps were not thresholded.

## RESULTS

### Behavioral Results

**Figure 2:**
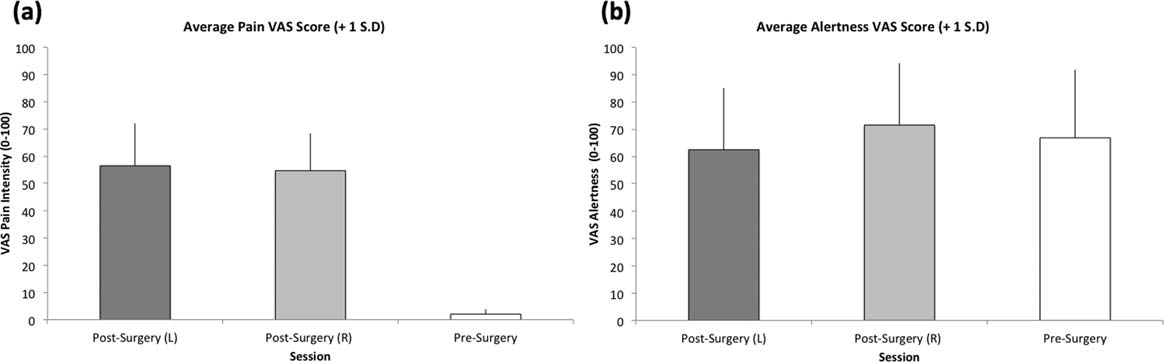
VAS indices indicate significant between‐session differences in perceived pain (**a**) but not in alertness (**b**).

As demonstrated in our previous work, session‐wise differences were identified in VAS indices of perceived pain (ANOVA: F = 111.62, df = 2,38, *P* < 0.0001) (Fig. 2a). Pair‐wise comparisons between sessions indicated that while left and right postsurgical states each differed from the average presurgical state (presurgical vs. postsurgical left‐Mean difference = 52.41, *P* < 0.0001; presurgical vs. postsurgical right‐Mean difference = 50.779, *P* < 0.0001), postsurgical left and right sessions did not differ from one another (Mean difference = 1.633, *P* = 1.00). There were no effects of session on VAS indices of alertness (ANOVA: F = 1.10, df = 2,38, *P* = 0.343) (Fig. 2b).

### GPC of Surgical Status

In the first analysis, we combined all ASL datasets from presurgical and postsurgical scanning sessions on left and right teeth to classify postsurgical from presurgical states. GPC correctly discriminated between states with 94.73% accuracy (classifier significance = *P* < 0.001).

Analysis (ii) assessed the performance of the GP classifier at separating left and right postsurgical images from each other. Classifier accuracy was 52.5%, just above chance and not significantly predictive (*P* > 0.439).

**Figure 3:**
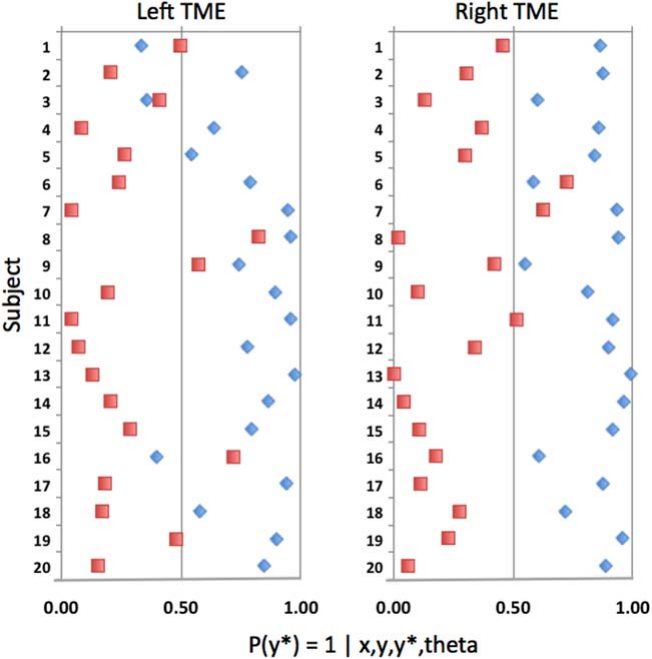
Predictive probabilities for ASL images collected at rest and ASL images collected postsurgically for both right and left extraction. The *x*‐axis indicates the probability that each scan was derived from the presurgery condition. Red squares indicate predictions for images collected postsurgery and are classified correctly if they have a predictive probability less than 0.5 (vertical black dashed line). The blue diamonds indicate the perfusion images acquired from the presurgery scanning session and are correctly classified if they have a predictive probability greater than 0.5. [Color figure can be viewed in the online issue, which is available at http://wileyonlinelibrary.com.]

In analysis (iii), we assessed classification accuracy between ASL images acquired during the pain‐free follow‐up session (S6) and postsurgical states for left and right sides separately, to mimic the constraints of a cross‐over trial design. When TME was performed on the left side, postsurgical ASL data could be discriminated from the pain‐free follow‐up session (Fig. 1a) with 85% accuracy (*P* < 0.001); and for the right side classification accuracy was 93% (*P* < 0.001). Figure 3 illustrates the GPC predictive probabilities for each individual subject in the cohort, of being in either presurgical or postsurgical states.

**Figure 4:**
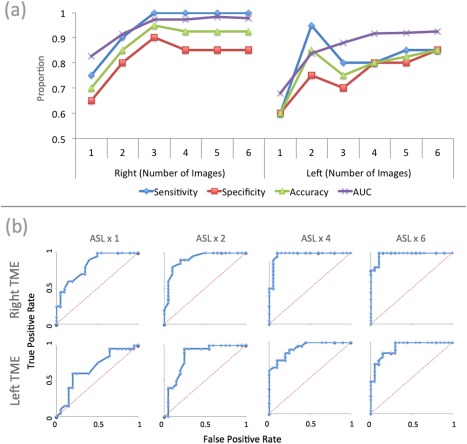
Accuracy, sensitivity, and specificity of the GP classifiers are demonstrated in (**a**) as a function of the number of ASL volumes (per subject and session) used for training the classifier. Area under the curves (AUC) is also shown with the respective ROC curves shown in (**b**) for classifiers trained with 1, 2, 4, and 6 images to demonstrate the stability of the ROC curves. [Color figure can be viewed in the online issue, which is available at http://wileyonlinelibrary.com.]

**Figure 5:**
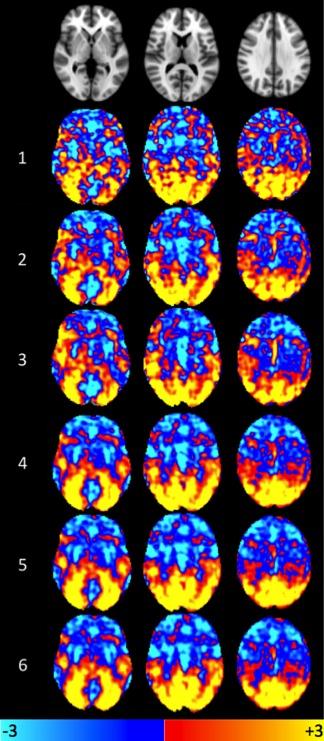
Representative GPC image patterns that separate left postsurgical vs. follow‐up/no‐surgery GP states. The top row provide anatomical slice locations in MNI template space. Lower rows illustrate the GP pattern discerned using 1 to 6 images to train the classifier. (Blue‐light blue colormap = negative GPC voxelwise coefficients favor postsurgical scans; Red–yellow colormap = positive GPC voxelwise coefficients favor no‐surgery classification).

We performed a stepwise removal of ASL images for calculation of the GPC to assess the impact of the number of images on GPC performance. In each iteration of this analysis, the final ASL image, in reverse acquisition order, was removed and the GPC retrained. Utilising two or more ASL images to inform the classifier resulted in significant (*P* < 0.01) discrimination of the pain‐free state at follow‐up from postsurgery states on both left and right sides with an accuracy of 80% or greater. Using only the first ASL image acquired did not result in significantly accurate classification of either left or right‐sided postsurgical state. We visualized the effects of systematic data reduction on classification accuracy as a simple line segment plot (Fig. 4a), which illustrated the “elbow” of the accuracy plot at the point on the *x*‐axis when only two ASL images were used to inform GP classification. The additional ASL data did not substantially increase classification accuracy. Receiver operating characteristic curves provided in Figure 4b demonstrate the effects of acquiring multiple ASL datasets on classification sensitivity and specificity. Classification accuracies and significances for all tests in analysis three are summarized in Table 1. Figure 5 indicates the spatial pattern of the GPC, or “g‐map” that discriminates between images derived from postsurgical and pain‐free follow‐up states. Each row in Figure 5 illustrates the effect of increasing the number of images available to the classifier to discriminate between postsurgical against pain‐free states. The magnitude of the GPC coefficient at each voxel provides a measure of the relative difference in activation between classes in the context of the entire pattern and the sign indicates (“favors”) the class with greater mean rCBF. In common with our previous report [Howard et al., 2011], the pattern of GPC coefficients favoring classification of the postsurgical condition (colored in blue) include the bilateral thalamus, posterior and anterior insula, secondary somatosensory, and anterior cingulate cortices; by contrast, the pattern favoring classification of the no surgery condition (colored in red–yellow) predominantly featured the occipital and posterior parietal cortices. For brevity, we have illustrated only the classifier for the left side; however, the spatial distribution of GPC coefficients was similar on the right.

**Table 1:** Categorical prediction accuracy for the classifiers for postextraction pain states against presurgical states using all scans (i)

*N* images	True positive	True negative	Accuracy	*P*‐Values
(i) All Presurgery vs. Postsurgery				
12	0.95	0.95	0.95	0.001
(ii) Follow‐up vs. Postsurgery (Left)				
1	0.6	0.6	0.6	0.17
2	0.95	0.75	0.85	0.001
3	0.8	0.7	0.75	0.01
4	0.8	0.8	0.8	0.001
5	0.85	0.8	0.825	0.001
6	0.85	0.85	0.85	0.001
(iii) Follow‐up vs. Postsurgery (Right)				
1	0.75	0.65	0.7	0.02
2	0.9	0.8	0.85	0.001
3	1	0.9	0.95	0.001
4	1	0.85	0.925	0.001
5	1	0.85	0.925	0.001
6	1	0.85	0.925	0.001

Classifier performance and significance is demonstrated as a function of the number of ASL images used per subject for the left (ii) and right (iii) postsurgical versus follow‐up scans.

## DISCUSSION

We have demonstrated accurate discrimination of on‐going postsurgical pain from pain‐free states in the same individuals. Classification of pain‐states did not rely on self‐report: instead, multivariate pattern classification (MPC) was performed on rCBF endpoints derived from ASL‐MRI. The technique is rapid, efficient, and ethical, minimising the amount of time patients suffering pain must endure the MRI experience. 80% classification accuracy or greater could be derived from as few as two ASL scans, equivalent to a total scanning session of approximately 15 min. Classification performance was similar for both left and right‐sided postsurgical pain. Although the multivariate pattern of brain regions underlying classification was physiologically plausible, compared with previous MRI investigations [Wasan et al., 2011], including a mass‐univariate analysis of a subset of these data [Howard et al., 2011], it is important to note that it is the multivariate pattern in these data that is being used to perform classification [Rosa and Seymour, 2014]. Two key benefits highlight the added value of pattern recognition over conventional analysis methods: first, they enable predictions regarding the condition of single individuals and the particular application of GPC provides accurate quantification of the predictive confidence of each of those individual predictions; second, the classifier results are generalizable, in the sense that they can be used to predict pain‐states in individual new patients. This work adds considerably to the field, by demonstrating its suitability to real‐world, clinically relevant functional indices of on‐going pain. In the discussion that follows we highlight the potential impact of the ASL/GPC methodology as a focussed, personalized approach to developing much‐needed new therapies.

It is clear that there is no difficulty in discriminating the presurgical from postsurgical states, either using neuroimaging or conventional self‐reported endpoints. That said, the TME model provides an ideal test‐bed for the development of ASL as a robust, ethical means of eliciting on‐going pain secondary to tissue trauma [Howard et al., 2011; Kupers and Kehlet, 2006; Tracey and Johns, 2010] and for testing the sensitivity and specificity of the GPC technique to detect inescapable on‐going pain. TME remains the gold‐standard model for making go/no‐go decisions in early analgesic development for many novel pharmacological entities, acting as a bridge between acute and persistent pain states [Dionne et al., 2005; Kupers and Kehlet, 2006; Tracey and Johns, 2010]. While up to 80% of individuals continue to experience considerable unwanted postsurgical pain [Apfelbaum et al., 2003], arguably the major challenge of developing new treatments lies with persistent pain states [Borsook et al., 2011]. The difficulty with these conditions is that it is still unclear whether neuroimaging markers directly predict the underlying clinical pathology, or represent neuroplastic maladaptive alterations in central nervous system functioning, likely sequelae of living with long‐term pain [May, 2008]. The application of MPC methodology applied to rCBF indices of on‐going pain is equally suitable to both acute and persistent pain states, but the feasibility of whether a “one‐size fits all” classifier, that universally discriminates between all pain states, will require considerable further investigation [Rosa and Seymour, 2014].

Reports describing conventional mass‐univariate approaches to studying pain using ASL, provide encouraging reading for future pain and therapeutic research [Rosa and Seymour, 2014]. These studies have demonstrated rCBF increases in patients with persistent pain secondary to osteoarthritis (OA), compared to controls [Howard et al., 2012], following augmentation of low back pain [Wasan et al., 2011], postherpetic neuralgia [Liu et al., 2013] and persistent trigeminal pain phenotypes [Youssef et al., 2014] indicating the sensitivity of the ASL technique to detect pain secondary to pathology. While this discussion focuses on ASL, other investigations using resting‐state fMRI have also detected differences in connectivity between brain regions in patients with persistent pain [Baliki et al., 2006; Napadow et al., 2010], as well as using rapid ASL‐derived indices of functional connectivity [Loggia et al., 2012].

Potentially these clinical investigations may also be used in concert with MPC techniques to provide diagnostic/prognostic predictions but to the best of our knowledge these studies have yet to be performed. Reports of MPC analyses in the field of pain research have largely focussed on conventional “evoked‐response” BOLD fMRI, reporting discrimination between experimentally induced painful and nonpainful stimulation using laser, electric, and thermal stimulations (reviewed in [Rosa and Seymour, 2014]) including predicting magnitude of subjective responses [Marquand et al., 2010]. Recently the generalizability of MPC analysis has been demonstrated [Wager et al., 2013] in experimental pain studies; whereby a “neurological signature” of pain derived from one study and sample could be used to predict acute experimental pain in other samples and experimental designs [Apkarian, 2013]. Early reports describing MPC applied to structural MRI endpoints [Baliki et al., 2011; Ung et al., 2014] are also encouraging for diagnostic purposes but arguably represent the downstream anatomical sequelae of persistent pain as opposed to pain per se. Importantly, these reports did not examine on‐going, clinically relevant pain as investigated here. On‐going pain is often the endpoint utilized in testing treatment efficacy [Tracey and Johns, 2010], leading us to suggest that the ASL+ML methodology has potential utility as an adjunct to established self‐report measures in the pursuit of new treatments. We stress that individualized prediction of treatment responses need not be limited to pharmacotherapies. By contrast, these MPC techniques should be equally applicable to predicting treatment responses to physical and psychological therapies in patients with persistent pain. We have previously argued that the provision of a neuroimaging biomarker that simply reproduces the information of self‐report, although neurobiologically interesting, is less important in the context of clinical trials. The MPC approach here is attempting to classify relative categories or states. The patterns, therefore, are not necessarily informative about within‐category states (e.g., how much pain an individual was in during a specific ASL scan), so provide information over and above pain ratings.

Outside the area of pain research, MPC techniques applied to MRI endpoints have demonstrated sensitivity to discriminate between individuals with other chronic neurological and psychiatric conditions, for example, depression, schizophrenia, Parkinson's, and Alzheimer's diseases (reviewed in [Orru et al., 2012]). MPC analyses on ASL data have also been used to discriminate accurately between drug classes in healthy volunteers [Marquand et al., 2012] and predict responses to drug treatments in patients with depression [Gong et al., 2011]. These reports indicate that the stage is set for the potential application of these techniques as diagnostic and prognostic tools in persistent pain states.

There are additional arenas in which the ASL/GPC technologies might be exploited, particularly where self‐reported pain is not possible. This is especially clear in preclinical pain research. Behavioral indices of pain (e.g., tail flicking, paw withdrawal turning/biting etc.) have been utilized, largely in the absence of an alternative mechanism for measuring pain [Mogil and Crager, 2004]. The availability of ASL on dedicated preclinical imaging systems makes translational research an enticing prospect. The addition of predictive MPC analysis techniques, however, adds further scope. Ordinal regression and multiclass pattern classification techniques are attractive propositions, to examine discrimination of novel compounds against those with of known efficacy (or proven lack of efficacy), differing mechanisms of action or examination of dose‐response rates [Doyle et al., 2013; Marquand et al., 2012]. A more efficacious marker of pain in animals especially would provide an earlier go/no‐go to clinical experiments, sparing both animals and later human trials. Outside the domain of therapeutic development, the MPC methodology provides the possible framework to make single‐subject predictions about whether someone is experiencing pain. Hence, this technology may be beneficial for patients unable to verbalise, for example, individuals with consciousness disorders [Owen and Coleman, 2008] or children. This may be particularly important in the case of the latter, where experience of pain in early development has been shown to have detrimental effects in later life [Liossi and Fitzgerald, 2012]. However, MPC techniques are not the panacea for these complex problems. This data‐driven approach to analysis strongly depends on appropriate and rigorous study design. Confounds, systemic or otherwise, introduced in a study design cannot be recorded or tested using multivariate classification methods in as flexible a fashion as in (e.g.,) general linear models [Todd et al., 2013].

In summary, we have demonstrated how state of the art MPC methods can be applied to ASL data to provide single‐subject predictions regarding the probability of an individual experiencing moderate to severe postoperative on‐going pain. The acquisition phase relies only on the consent of the individual, is rapid and requires no other experimental intervention. Importantly, the output of MPC can be generalized to new subjects. These findings represent tentative, but vital, steps toward the goals of personalized medicine in health care and evaluation of novel therapeutics. These methods must now be applied in two areas, examining individuals with persistent pain, and predicting prognostic outcomes. We contend that neuroimaging may, finally, be taking steps toward providing a complementary measure to self‐report for pain measurement.
